# Analysis of the predictive value of 3-day cumulative energy deficit for 28-day mortality in patients with sepsis and nutritional risk: a retrospective study

**DOI:** 10.3389/fnut.2026.1749483

**Published:** 2026-06-04

**Authors:** Haodi Luan, Qianqian Liu, Hua Fan, Yahui Guo, Chenxi Cao, Jing Zhao, Jing Lin

**Affiliations:** 1 Department of Critical Care Medicine, The Affiliated Hospital of Inner Mongolia Medical University, Hohhot, China; 2 Department of Clinical Nutrition, The Affiliated Hospital of Inner Mongolia Medical University, Hohhot, China; 3 Department of Gastroenterology, The Affiliated Hospital of Inner Mongolia Medical University, Hohhot, China

**Keywords:** 28-day mortality rate, cumulative energy deficit, nutritional risk, risk factors, sepsis

## Abstract

**Objective:**

Cumulative energy deficit in the early phase of critical illness is associated with increased mortality and worsened clinical outcomes. To identify early risk factors for 28-day mortality in patients with sepsis combined with nutritional risk to inform early clinical recognition and intervention.

**Methods:**

A retrospective analysis of clinical data from sepsis patients in the ICU at the Affiliated Hospital of Inner Mongolia Medical University was conducted from January 2022 to January 2025 (ethics approval: KY2025177). Patients were categorized as survivors or non-survivors based on 28-day outcomes. Nutritional risk was defined as NRS-2002 score ≥ 3. Sepsis was diagnosed according to Sepsis-3.0 criteria. The 3-day cumulative energy deficit was calculated as the difference between target energy requirement (estimated using Harris-Benedict formula with 25 kcal/kg coefficient) and actual energy intake (enteral, parenteral, and oral) over the first 3 days of ICU admission. Risk factors were identified through univariate and multivariate logistic regression, and their predictive effectiveness was evaluated using ROC curves. Subgroup analyses employed optimal cutoff values.

**Results:**

Among 516 eligible patients, 360 were analyzed (305 survivors and 55 non-survivors). The median age was 70 years (IQR 58.0–77.0) in the survival group and 74 years (IQR 62.0–85.0) in the death group (*p* = 0.003). Males accounted for 60.98% (186/305) in the survival group and 72.73% (40/55) in the death group. The median NRS-2002 score was 5.0 (IQR 4.0–6.0) in both groups, median APACHE II score was 18.0 (IQR 15.0–20.0) vs. 22.0 (IQR 20.0–25.0), and median SOFA score was 6.0 (IQR 5.0–8.0) vs. 8.0 (IQR 7.5–10.0) in survivors vs. non-survivors, respectively (all *p* < 0.05). The 28-day mortality after ICU admission was 15.28% (55/360). Multivariate logistic regression identified age (OR 1.061, 95% CI: 1.032–1.091, *p* < 0.001) and 3-day cumulative energy deficit (OR 1.002, 95% CI: 1.001–1.002, *p* < 0.001) as independent risk factors for 28-day mortality. ROC curve analysis confirmed 3-day cumulative energy deficit had predictive value, with an AUC of 0.881 (95% CI: 0.835–0.927). Subgroup analysis using the cutoff of ≥2,916 kcal showed patients with higher energy deficit had significantly higher SOFA, APACHE II, NRS2002 scores and higher mortality (*p* < 0.05).

**Conclusion:**

This study identified age and 3-day cumulative energy deficit as early independent predictors of 28-day mortality in septic patients with nutritional risk. A cumulative energy deficit threshold of ≥2,916 kcal over 3 days demonstrated discriminative value for risk stratification. These findings underscore the prognostic significance of early energy balance monitoring in this high-risk population and may inform the design of future interventional studies targeting nutritional optimization.

## 1 Introduction

Sepsis is a common critical illness in intensive care units, characterized by systemic inflammatory response, immune dysfunction, microcirculatory issues, and multi-organ failure. These factors lead to high mortality rates and poor prognoses for patients (1). Nutritional risk is a significant complication in critically ill patients, with a prevalence ranging from 38 to 78% depending on the assessment tool and population studied (2). Among patients with sepsis specifically, the prevalence of malnutrition risk is reported to be 47.5% (3). The interplay between sepsis and malnutrition creates a vicious cycle: sepsis-induced hypermetabolism increases energy expenditure and protein breakdown, exacerbating malnutrition (4). Conversely, malnutrition-induced immunosuppression impairs immune cell function and increases infection susceptibility (5).

Protein-energy malnutrition induces profound immunosuppression through thymic atrophy, reduced T-cell activation, and impaired antigen-presenting cell function (6). Severe malnutrition is associated with significant thymic atrophy, decreased leptin levels, and altered cytokine profiles, creating a state of secondary immunodeficiency (7). Hypoalbuminemia, a marker of malnutrition, is associated with systemic hyperinflammatory state and endothelial dysfunction (8). Inflammation further suppresses albumin synthesis through increased capillary leakage and reduced hepatic production, creating a bidirectional interaction that exacerbates both conditions (9). This confluence of immunosuppression and unabated inflammation creates a “perfect storm” predisposing patients to multi-organ dysfunction (10).

There is a notable lack of sensitive indicators for assessing the prognosis of sepsis patients at nutritional risk. Cumulative energy deficit has emerged as a potential prognostic marker in critically ill patients (11). The first 72 h represent a critical metabolic window following ICU admission, during which energy deficit accumulation is most pronounced (12). According to the 2023 ESPEN guidelines, progressive energy delivery should achieve at least 70% of needs within days 3–7 (13). This suggests that the first 3 days constitute a distinct prognostic phase. Shorter windows (e.g., 24 h) fail to capture cumulative metabolic burden (14), whereas longer windows (e.g., 7 days) introduce confounding from clinical management variations and metabolic phase transitions (15). Therefore, this study investigates the predictive value of 3-day cumulative energy deficit for 28-day mortality in ICU patients with sepsis and nutritional risk.

## 2 Methods

### 2.1 Ethics statement

The study was approved by the Ethics Committee of the Affiliated Hospital of Inner Mongolia Medical University (approval number: KY2025177) with a waiver of individual informed consent, and registered on ClinicalTrials.gov (registration number: NCT07278167). The study data were extracted from the hospital’ s pre-established patient database, for which written informed consent had been obtained from all patients during database construction, and all data were anonymized and de-identified in compliance with ethical regulations.

### 2.2 Study design and participants

From May 2025 to July 2025, we accessed the Medu Cloud database of the Affiliated Hospital of Inner Mongolia Medical University to extract relevant data indicators covering the period from January 2022 to January 2025. If the patients had been admitted to ICU several times, only the clinical data of the first ICU admission were collected. Inclusion criteria: (1) met the diagnostic criteria for sepsis 3.0 (16); (2) met the diagnostic criteria for nutritional risk (NRS-2002 score ≥ 3) (17); (3) ICU stay ≥ 2 d; (4) age ≥ 18. Exclusion criteria: (1) edema (defined as generalized edema or edema affecting nutritional assessment, as it may interfere with accurate body weight measurement and nutritional status evaluation); (2) tumor patients (defined as ongoing cancer treatment, uncontrolled disease, or diagnosis within 6 months prior to ICU admission); (3) pregnancy; (4) lack of complete laboratory results. The included patients were categorized into survival and death groups based on their clinical outcomes within 28 days of ICU admission. Laboratory indicators were obtained within 24 h of ICU admission.

### 2.3 Assessment of target energy requirement and actual energy intake

Target energy requirement of the enrolled ICU patients was estimated using the Harris-Benedict Estimation of Basal Energy Expenditure (BEE) formula with a standardized coefficient of 25 kcal/kg. This method was selected primarily due to the presence of non-mechanically ventilated patients in the baseline cohort, who were ineligible for indirect calorimetry due to technical and clinical constraints. For the assessment of actual energy intake (AEI), all data were collected and verified by certified clinical dietitians from the hospital. AEI included three major components: parenteral energy intake, enteral energy intake, and oral dietary energy intake. All energy intake values were quantified and expressed in kilocalories (kcal) to ensure consistency and comparability of the data.

### 2.4 Outcome measures

Collect the following baseline patient data: age, sex, vital signs, smoking and alcohol histories, history of underlying diseases (hypertension, diabetes, heart failure, respiratory, gastrointestinal, genitourinary, endocrine, neurologic, hematologic, musculoskeletal, and rheumatic/immune disorders); nutritional parameters including height, weight, BMI, and 3-day cumulative energy deficit, which was calculated over the first 3 days of ICU admission; APACHE II, SOFA, and NRS2002 scores; duration of mechanical ventilation; ICU length of stay; mortality; and laboratory values from the first assessment within 24 h after ICU admission.

### 2.5 Statistical analyses

Statistical analyses were carried out with SPSS 22.0. Normality and homogeneity of variance were tested for all variables. Continuous variables following normal distribution are expressed as mean ± SD, and non-normally distributed continuous variables are expressed as median with interquartile range [Median (P25, P75)]. Between-group comparisons for normally distributed continuous data were performed using the independent-samples t-test, while non-normally distributed continuous data were analyzed by Mann–Whitney *U* tests, and categorical variables are expressed as n (%) and were compared with the *χ*^2^ test. Patient clinical outcome served as the dependent variable. Variables that were statistically significant in the univariate analysis were introduced into a multivariate binary logistic regression model to determine independent predictors. Significant predictors identified by the regression were further subjected to ROC curve analysis, where the AUC quantified prognostic discrimination for severe cases, to evaluate their prognostic performance in severe cases and to determine optimal cut-off values via the Youden index. A two-sided *p* < 0.05 was considered statistically significant.

## 3 Results

### 3.1 Baseline characteristics

**Figure 1:**
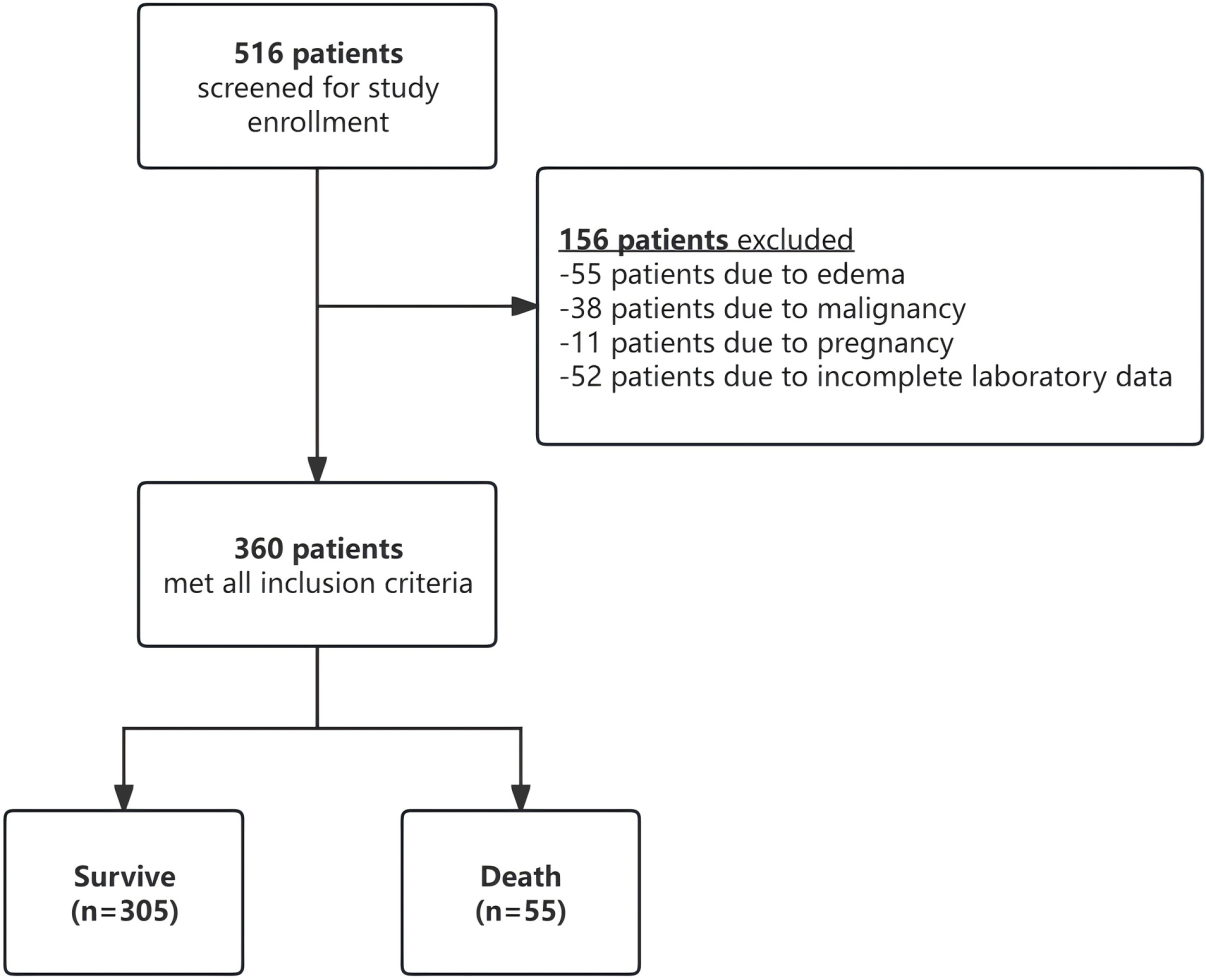
Study flowchart.

Of the 516 eligible patients, 156 were excluded due to oedema (55), tumors (38), pregnancy (11), and incomplete laboratory results (52), resulting in 360 patients for analysis. Based on the clinical outcomes at day 28 after ICU admission, patients were divided into a survival group (305) and a death group (55) (Figure 1). The median age in the mortality group was 74 years, significantly higher than 69 years in the survival group (*p* = 0.003). The majority of patients were male, with 60.98% in the mortality group and 72.73% in the survival group. Median ICU length of stay was longer in the mortality group at 10.6 days compared to 7 days in the survival group (*p* = 0.034). Non-survivors had higher APACHE II, SOFA, and NRS2002 scores upon ICU admission and longer mechanical ventilation duration (all *p* < 0.05). The cumulative energy deficit over 3 days was 3,900 kcal in the mortality group, higher than the 3,000 kcal in the survival group (*p* < 0.05). No significant differences were noted in gender, smoking history, alcohol use, or underlying conditions (*p* > 0.05) (Table 1 and Appendix Table S1). Beyond the demographic differences, non-survivors presented with markedly higher disease severity upon ICU admission. The median APACHE II score was 4 points higher in the death group than in the survival group (22.0 vs. 18.0), and the SOFA score was 2 points higher (8.0 vs. 6.0), both indicating substantially greater organ dysfunction burden in non-survivors (*p* < 0.001 for both). Notably, the 3-day cumulative energy deficit was 900 kcal greater in non-survivors compared with survivors (median 3,900 kcal vs. 3,000 kcal), representing a 30% relative increase in energy debt (*p* < 0.001). The ICU length of stay was also 3.6 days longer in non-survivors (10.6 days vs. 7.0 days, *p* = 0.034), suggesting prolonged critical illness course in patients with higher energy deficits.

**Table 1:** Baseline characteristics of the study participants (*N* = 360).

Characteristic	28-day clinical outcomes		*p*
	Survive (*n* = 305)	Death (*n* = 55)	
Demographics			
Age, years, median [IQR]	69.000(58.0,77.0)	74.000(62.0,85.0)	0.003**
Male, sex, *n* (%)	186(60.98)	40(72.73)	0.097
Nutritional parameters			
Height, cm, median [IQR]	168.000(160.0,173.0)	170.000(167.0,174.5)	0.053
Weight, kg, median [IQR]	65.000(55.0,72.1)	65.000(52.5,70.0)	0.479
BMI, median [IQR]	22.885(20.0,25.4)	21.914(19.0,23.9)	0.094
3-Day Cumulative Energy Deficit	3000.000(1500.0,3900.0)	3900.000(2550.0,4200.0)	<0.001**
Disease severity scores			
APACHE II score, median [IQR]	18.000(15.0,20.0)	22.000(20.0,25.0)	<0.001**
SOFA score, median [IQR]	6.000(5.0,8.0)	8.000(7.5,10.0)	<0.001**
NRS2002 score, median [IQR]	5.000(4.0,6.0)	6.000(4.5,6.0)	0.018*
Laboratory indices			
WBC, ×10^9^/L, median [IQR]	11.050(7.7,15.6)	9.990(6.4,14.5)	0.132
RBC, ×10^12^/L, mean ±SD	3.965(3.3,4.5)	3.380(2.7,4.1)	<0.001**
HGB, g/L, mean ±SD	120.43 ± 30.04	105.78 ± 26.25	0.001**
PLT, ×10^9^/L, median [IQR]	182.000(123.0,247.0)	144.000(95.0,189.0)	0.006**
CRP, mg/L, median [IQR]	79.500(29.3,151.4)	79.400(28.6,157.3)	0.567
PCT, ng/mL, median [IQR]	0.597(0.1,3.7)	1.286(0.4,6.6)	0.021*
ALT, IU/L, median [IQR]	27.000(15.0,54.0)	23.000(12.5,50.8)	0.247
AST, IU/L, median [IQR]	33.000(20.9,66.2)	41.200(24.0,71.0)	0.216
TBIL, umol/L, median [IQR]	15.900(10.1,28.1)	15.000(10.7,24.9)	0.967
ALB, g/L, mean ±SD	30.900(27.4,35.3)	29.200(25.0,33.3)	0.068
PA, mg/dL, median [IQR]	10.900(6.5,16.2)	8.500(4.5,13.2)	0.036*
CREA, umol/L, median [IQR]	72.000(51.5,112.5)	82.000(58.0,237.0)	0.044*
PT, second, median [IQR]	13.200(12.0,14.7)	14.000(12.8,15.6)	0.002**
APTT, second, median [IQR]	29.000(26.6,32.7)	33.200(28.2,36.3)	<0.001**
INR, median [IQR]	1.160(1.1,1.3)	1.270(1.1,1.4)	0.002**
FIB, g/L, median [IQR]	4.280(2.8,5.9)	3.600(1.8,5.0)	0.019*
Ventilator days, median [IQR]	2.580(0.6,6.0)	3.560(1.0,8.9)	0.036*
ICU hospitalization days, median [IQR]	7.000(3.5,13.4)	10.600(4.5,19.0)	0.034*

*p < 0.05, **p < 0.01.

Data are number (%), mean ± standard deviation or median [interquartile range].

APACHE II, Acute Physiology and Chronic Health Evaluation II; SOFA, Sequential Organ Failure Assessment; NRS2002, Nutritional Risk Screening 2002; BMI, body mass index; IQR, inter-quartile range; SD, standard deviation; WBC, white blood cell count; RBC, red blood cell count; HGB, hemoglobin; PLT, platelet count; CRP, C-reactive protein; PCT, procalcitonin; ALT, alanine aminotransferase; AST, aspartate aminotransferase; TBil, total bilirubin; ALB, albumin; PA, prealbumin; Crea, creatinine; PT, prothrombin time; APTT, activated partial thromboplastin time; INR, international normalized ratio; FIB, fibrinogen.

### 3.2 Laboratory parameter analysis

Laboratory indices revealed distinct metabolic and organ dysfunction profiles between survivors and non-survivors (Table 1 and Appendix Table S1). Non-survivors exhibited significantly more pronounced markers of infection severity and coagulopathy: procalcitonin levels were more than doubled (median 1.286 ng/mL vs. 0.597 ng/mL, *p* = 0.021), and creatinine was elevated (median 82.0 vs. 72.0 μmol/L, *p* = 0.044), suggesting greater septic burden and acute kidney injury in the death group. Hematological parameters indicated worse nutritional status and bone marrow suppression in non-survivors: hemoglobin was 14.65 g/L lower (105.78 vs. 120.43 g/L, *p* < 0.001), platelet count was reduced by 38 × 10^9^/L (144.0 vs. 182.0, *p* = 0.006), and prealbumin was markedly decreased (median 8.5 vs. 10.9 mg/dL, *p* = 0.036). Coagulation profiles were substantially prolonged in non-survivors, with PT extended by 0.8 s (14.0 vs. 13.2 s, *p* = 0.002), APTT prolonged by 4.2 s (33.2 vs. 29.0 s, *p* < 0.001), and INR elevated (1.27 vs. 1.16, *p* = 0.002), alongside lower fibrinogen levels (3.60 vs. 4.28 g/L, *p* = 0.019). These findings collectively indicate that non-survivors presented with more severe sepsis-related organ dysfunction, worse nutritional reserve, and greater coagulation disturbances at ICU admission.

### 3.3 Logistic regression analysis

In a multivariate logistic regression analysis, statistically significant indicators from the univariate analysis—including age, cumulative energy deficit over 3 days, PCT, CREA, PT, APTT, INR, RBC, HGB, PLT, PA, and FIB—were employed as independent variables, with clinical death as the dependent variable. The results demonstrated that increased age and cumulative energy deficit over 3 days constitute independent risk factors for clinical death (*p* < 0.05) (Table 2).

**Table 2:** Summary of binary logit regression analysis results.

Characteristic	OR	95% CI	Regression coefficient	*p*
Age	1.061	1.032 ~ 1.091	0.059	<0.001**
3-day cumulative energy deficit	1.002	1.001 ~ 1.002	0.002	<0.001**
RBC	0.739	0.270 ~ 2.020	−0.302	0.556
HGB	0.996	0.963 ~ 1.030	−0.004	0.803
PLT	0.996	0.991 ~ 1.000	−0.004	0.073
PCT	0.998	0.980 ~ 1.017	−0.002	0.841
PA	1.004	0.962 ~ 1.047	0.004	0.857
PT	1.001	0.917 ~ 1.093	0.001	0.978
APTT	1.01	0.983 ~ 1.039	0.01	0.462
INR	0.898	0.369 ~ 2.185	−0.107	0.813
FIB	0.951	0.761 ~ 1.189	−0.05	0.661
CREA	1.001	0.999 ~ 1.003	0.001	0.184

RBC, red blood cell count; HGB, hemoglobin; PLT, platelet count; PCT, procalcitonin; PA, prealbumin; PT, prothrombin time; APTT, activated partial thromboplastin time; INR, international normalized ratio; FIB, fibrinogen; CREA, creatinine.

**p < 0.01.

### 3.4 ROC curve analysis

**Figure 2:**
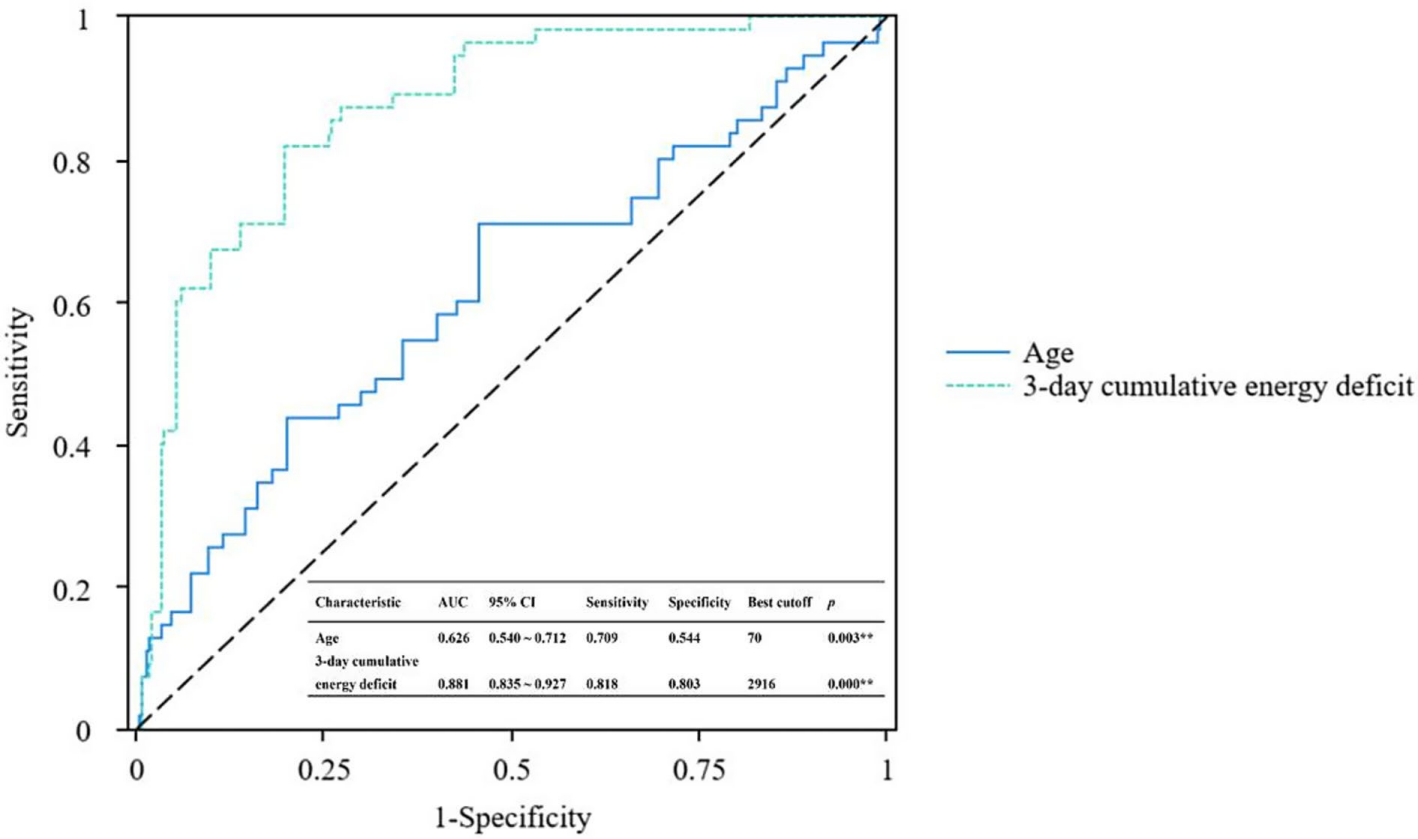
ROC analysis.

The area under the ROC curve (AUC) for predicting 28-day clinical mortality in patients with sepsis and nutritional risk was 0.626 (95% CI: 0.540–0.712) for age and 0.881 (95% CI: 0.835–0.927) for cumulative energy deficit over 3 days. The optimal age cutoff was 70 years, yielding a sensitivity of 70.9% and specificity of 54.4% for predicting 28-day mortality of ICU admission in patients with sepsis and nutritional risk. The optimal cut-off value for cumulative energy deficit over 3 days was 2,916 kcal, yielding a sensitivity of 81.8% and specificity of 80.3% for predicting clinical death within 28 days of ICU admission in patients with sepsis and nutritional risk (Figure 2). The logistic regression model demonstrated satisfactory goodness-of-fit according to the Hosmer–Lemeshow test (*χ*^2^ = 5.899, *df* = 8, *p* = 0.659) (Table 3).

**Table 3:** Hosmer–Lemeshow test.

*χ* ^2^	*df*	*p*
5.899	8	0.659

### 3.5 Subgroup analysis on the cutoff value for 3-day cumulative energy deficit

**Figure 3:**
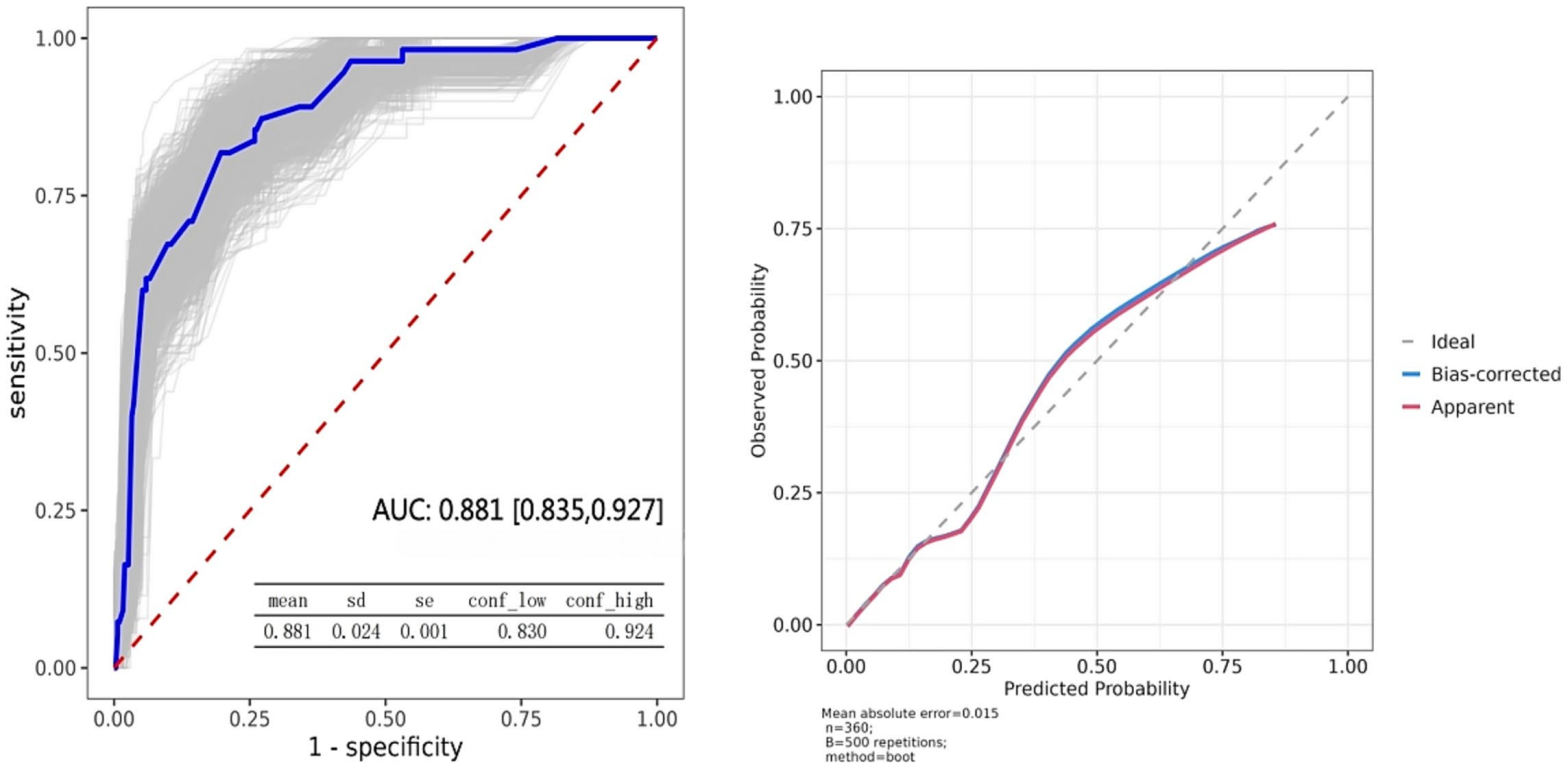
Bootstrap validation and calibration analysis.

**Figure 4:**
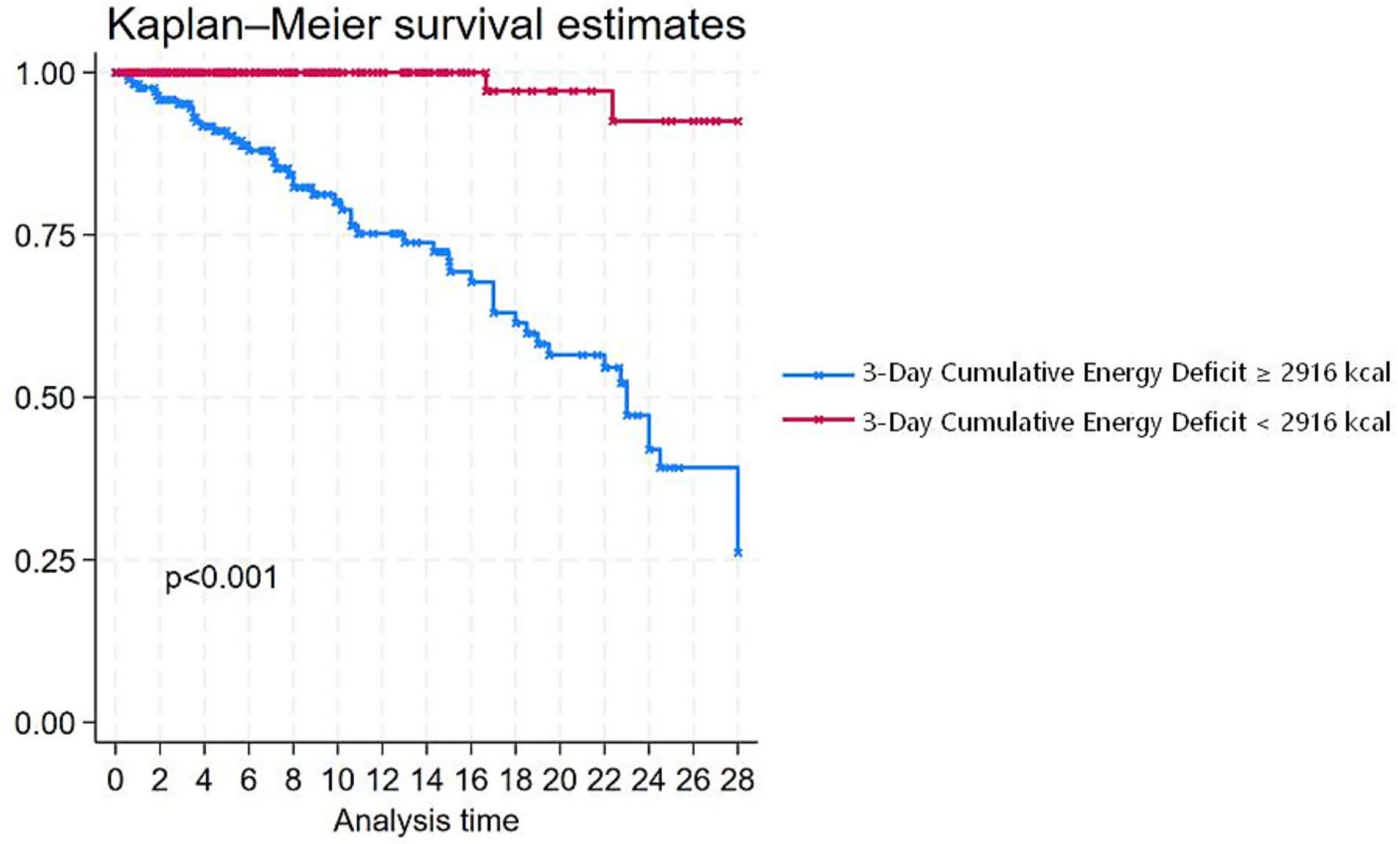
Kaplan-Meier survival analysis.

Given the low AUC value for age observed in the ROC regression analysis, a subgroup analysis was conducted focused on the optimal cut-off value for 3-day cumulative energy deficit. To account for potential inherent bias, the ROC curve for 3-day cumulative energy deficit was validated through 1,000 bootstrap repetitions, yielding a bootstrap-corrected AUC of 0.857 (95% CI: 0.799, 0.912), which demonstrated statistical power comparable to that of the initial model. Additionally, calibration curve analysis was performed to assess the agreement between predicted and observed probabilities. The apparent and bias-corrected curves closely approximated the ideal diagonal line, with a mean absolute error of 0.015, indicating good calibration performance of this single predictor (Figure 3). Within this framework, a total of 360 patients diagnosed with sepsis and identified as having nutritional risk were categorized into two distinct groups. The results revealed that patients in the ≥2,916 kcal group exhibited significantly elevated SOFA scores, APACHE II scores, and 28-day mortality rates when compared to their counterparts in the <2,916 kcal group (*p* < 0.05). Although there were notable differences in NRS2002 scores between the groups (*p* < 0.05), the median values did not show significant variation (Table 4). Based on the respective cutoff values, we performed a survival analysis, the results showed that patients with ≥2,916 kcal groups exhibited lower survival rates (*p* < 0.001) (Figure 4).

**Table 4:** Subgroup analysis based on the cut-off point of 3-day cumulative energy deficit.

Characteristic	3-day cumulative energy deficit < 2,916 kcal (*n* = 254)	3-day cumulative energy deficit≥2,916 kcal (*n* = 106)	*Z*/*χ*^2^	*p*
Sofa	6.000(5.0,8.0)	7.000(5.0,8.0)	−2.909	0.004**
APPACHE II	18.000(15.0,20.0)	20.000(18.0,25.0)	−3.913	0.000**
NRS2002	5.000(4.0,6.0)	5.000(4.0,6.0)	−2.522	0.012*
Mechanical ventilation duration (d)	2.690(0.5,6.0)	3.135(0.9,7.0)	−1.524	0.127
ICU duration of stay (d)	7.000(3.4,14.0)	7.960(3.9,17.0)	−1.028	0.304
ICU 28-day mortality rate	10(3.94)	45(42.45)	85.715	0.000**

*p < 0.05, ** p < 0.01.

SOFA, sequential organ failure assessment; APACHE II, acute physiology and chronic health evaluation II; NRS2002, nutritional risk screening 2002; ICU, intensive care unit.

## 4 Discussion

Early identification of risk factors in patients with sepsis and nutritional risk is essential to improve prognosis. Related studies have reported that the ICU 28-day mortality rate in patients with sepsis combined with nutritional risk can be 17.9–36.4% (5). The 28-day mortality rate of 15.28% in the present study may be associated with differences in study area, year, treatment, population baseline, and diagnostic criteria.

Energy deficit refers to the cumulative difference between a patient’s actual energy intake and their target energy requirements (18). In the early stages of sepsis, the body’s energy expenditure increases significantly due to hypermetabolic states, tissue hypoxia, and the effects of the cytokine storm (19). If energy intake is severely inadequate at this stage, it rapidly leads to the accumulation of energy deficit, thereby exacerbating protein breakdown, immune suppression, and multiple organ dysfunction (10). The term “3-day cumulative energy deficit” as defined in this study specifically denotes the accumulated energy shortfall within the 3 days after ICU admission, emphasizing its role as an early warning indicator.

Our multivariate analysis identified age and 3-day cumulative energy deficit as independent risk factors for 28-day mortality. Age had only modest predictive discrimination (AUC = 0.626), consistent with the well-known but nonspecific association between advancing age and poorer sepsis outcomes (20, 21). In contrast, the 3-day cumulative energy deficit showed excellent predictive performance (AUC = 0.881).

This finding aligns with previous studies on energy balance in sepsis but also extends them in important ways. Heidegger et al. demonstrated in a landmark RCT that supplemental parenteral nutrition initiated on day 3 in critically ill patients with insufficient enteral intake reduced nosocomial infections and improved clinical outcomes, supporting the concept that timely energy optimization is beneficial (22). Singer et al. (23), through the TICACOS trial, showed that tight calorie control aiming to match measured energy expenditure led to a trend toward lower mortality, reinforcing the notion that both underfeeding and overfeeding are detrimental in the acute phase. Our results are consistent with these studies in confirming that early energy deficit is a key driver of adverse outcomes. However, while Heidegger and Singer focused on the timing and adequacy of energy delivery relative to measured expenditure, our study provides a specific, quantifiable cumulative threshold (≥2,916 kcal over the first 3 days) that can be used for early bedside risk stratification—a practical tool not previously reported in the literature.

More recently, the evidence base has expanded considerably. A 2025 *post hoc* analysis of the multicenter NEED trial (*n* = 1,162 sepsis patients) demonstrated that achieving ≥60% of energy target within the first 7 days was associated with a 41.2% lower 28-day mortality risk in high-nutritional-risk patients (mNUTRIC ≥5; HR = 0.588, 95% CI: 0.388–0.891), while no survival benefit was observed in low-risk patients (24). These findings underscore the importance of risk-stratified nutritional strategies-a principle that resonates with our identification of a specific energy deficit cutoff. Another prospective observational study (*n* = 584) found that achieving energy sufficiency (≥70% of target) at 4–7 days post-ICU admission was associated with the lowest mortality (15.6% vs. 32.0% in the late group), suggesting an optimal window for nutritional intervention (15). Furthermore, a secondary analysis of a multicenter prospective study (*n* = 361) revealed a non-linear “hockey-stick” association between early (first 72 h) energy delivery and 28-day mortality: increasing energy delivery from 0 to 18 kcal/kg/day was associated with decreasing mortality (HR = 0.892), whereas delivery exceeding 18 kcal/kg/day was associated with increased mortality (HR = 1.116) in normal-BMI patients—cautions against both underfeeding and overfeeding in the early phase (25).

Collectively, these studies converge on the consensus that early energy deficit is a critical modifiable risk factor in sepsis. Our results specifically extend this body of evidence by: (1) providing a concrete, quantifiable cutoff (≥2,916 kcal over 3 days) for cumulative energy deficit that has not been previously established; (2) demonstrating that this metric outperforms static nutritional scores (NRS2002) in predicting 28-day mortality; (3) validating its excellent predictive performance (AUC = 0.881) in a cohort of septic patients with nutritional risk.

Notably, although the NRS2002 scores differed statistically between the two groups, the median values were identical (5.0 in both groups), suggesting that traditional nutritional risk screening tools may have limited sensitivity to early, dynamic metabolic disturbances (26). The NRS2002 was originally developed to detect chronic or subacute malnutrition and relies on static variables such as weight loss, reduced intake, and disease severity. It does not capture the rapid, progressive energy deficit that accumulates over hours to days in sepsis. In contrast, cumulative energy deficit is a continuous, quantifiable, and time-sensitive metric that directly reflects the ongoing mismatch between supply and demand during the hypermetabolic “storm phase” (27).

The clinical significance of this metric lies in its transcendence of traditional static scoring systems. A cutoff of ≥2,916 kcal can identify high-risk patients early, enabling proactive nutritional support—such as earlier initiation of enteral or parenteral nutrition—during the “golden window” before irreversible organ failure occurs. This shifts clinical focus to the emergency department or even pre-hospital stage, enabling early initiation of active nutritional therapy for high-risk patients and holding promise for reversing metabolic imbalance and improving outcomes (28).

This metric captures the cumulative energy deficit sustained by patients during the critical window from onset to ICU admission. Higher deficits (with ≥2,916 kcal as the optimal cut-off value) indicate a greater energy gap during the high-metabolic, high-catabolic “storm phase,” directly leading to more severe organ dysfunction (higher SOFA and APACHE II scores) and poorer clinical outcomes. This not only reveals energy debt as the pivotal link connecting early pathophysiological disruption to ultimate prognosis, but more importantly, it provides a clear early warning target and temporal window for implementing precise nutritional interventions during the “golden window period.” This shifts clinical focus to the emergency department or even pre-hospital stage, enabling the early initiation of active nutritional therapy for high-risk patients. This approach holds promise for reversing metabolic imbalance and improving outcomes.

## 5 Study limitation

Due to its retrospective, single-center study design, this research is subject to several limitations: Firstly, owing to missing relevant data, our diagnosis of nutritional risk relied solely on the NRS2002 score without incorporating the NUTRIC score tailored for ICU patients, and analysis of blood gas parameters was absent; Secondly, we only recorded the first measurement within 24 h of ICU admission, while subsequent measurements to observe dynamic changes might yield more meaningful conclusions, and current databases lack records of patients’ energy intake prior to ICU admission; Thirdly, the energy cutoff of 2,916 kcal requires external validation in multicenter cohorts to confirm its generalizability to other populations or settings; Fourthly, exclusion of 52 patients with incomplete data may have introduced selection bias, and the retrospective design precluded adjustment for competing risks and time-varying confounders. These limitations restrict the generalisability of our findings, and prospective studies are warranted to validate these results.

## Conclusion

This study identified age and 3-day cumulative energy deficit as early independent predictors of 28-day mortality in septic patients with nutritional risk. A cumulative energy deficit threshold of ≥2,916 kcal over 3 days demonstrated discriminative value for risk stratification. These findings underscore the prognostic significance of early energy balance monitoring in this high-risk population and may inform the design of future interventional studies targeting nutritional optimization.

## Data Availability

The original contributions presented in the study are included in the article/Supplementary material, further inquiries can be directed to the corresponding author.

## Glossary

ALB: Albumin
APACHE II: Acute Physiology and Chronic Health Evaluation II
APTT: Activated Partial Thromboplastin Time
ALT: Alanine Aminotransferase
ALP: Alkaline Phosphatase
AST: Aspartate Aminotransferase
AUC: Area Under the Curve
BUN: Blood Urea Nitrogen
BMI: Body Mass Index
CREA: Creatinine
CI: Confidence Interval
CRP: C-Reactive Protein
CT: Computed Tomography
FIB: Fibrinogen
GLB: Globulin
GLU: Glucose
GGT: Gamma-Glutamyl Transferase
HGB: Hemoglobin
ICU: Intensive Care Unit
IL-6: Interleukin-6
INR: International Normalized Ratio
MPV: Mean Platelet Volume
MRI: Magnetic Resonance Imaging
NEU: Neutrophil Count
NRS 2002: Nutritional Risk Screening 2002
OR: Odds Ratio
PA: Prealbumin
PLT: Platelet Count
PCT: Procalcitonin
PT: Prothrombin Time
RBC: Red Blood Cell Count
ROC: Receiver operating characteristic
SOFA: Sequential Organ Failure Assessment
TBIL: Total Bilirubin
TC: Total Cholesterol
TG: Triglycerides
UA: Uric Acid
WBC: White Blood Cell Count
