# Supplementary material for: Analysis of the predictive value of 3-day cumulative energy deficit for 28-day mortality in patients with sepsis and nutritional risk: a retrospective study
Source: Front Nutr. 2026 Jun 4;13:1749483. doi: 10.3389/fnut.2026.1749483 (PMC13275212; doi:10.3389/fnut.2026.1749483)
Supplement: Supplementary file 1 [file Table_1.DOCX]

| Baseline Characteristics of the Study Participants (N= 360) | | | |
| --- | --- | --- | --- |
| Characteristic | 28-day clinical outcomes | |  |
|  | Survive(n=305) | Death(n=55) | *p* |
| Comorbidities, n (%) |  |  |  |
| Hypertension | 146(47.87) | 28(50.91) | 0.678 |
| Diabetes | 80(26.23) | 17(30.91) | 0.472 |
| Heart failure | 94(30.82) | 21(38.18) | 0.281 |
| Pulmonary disease | 92(30.16) | 16(29.09) | 0.873 |
| Gastrointestinal disease | 111(36.39) | 19(34.55) | 0.793 |
| Renal disease | 62(20.33) | 9(16.36) | 0.496 |
| Endocrine disease | 22(7.21) | 3(5.45) | 0.854 |
| Hematologic disease | 8(2.62) | 0(0.00) | 0.473 |
| Musculoskeletal disease | 13(4.26) | 0(0.00) | 0.243 |
| Autoimmune disease | 18(5.90) | 2(3.64) | 0.722 |
| Site of infection |  |  |  |
| Pulmonary | 175(57.38) | 38(69.09) | 0.104 |
| Urinary Tract | 10(3.28) | 1(1.82) | 0.878 |
| Biliary Tract and Liver | 25(8.20) | 1(1.82) | 0.162 |
| Abdominal Cavity | 64(20.98) | 8(14.55) | 0.272 |
| Skin and Soft Tissue | 13(4.26) | 1(1.82) | 0.628 |
| Bloodstream | 1(0.33) | 0(0.00) | 1 |
| Nervous System | 1(0.33) | 0(0.00) | 1 |
| Multisystem Infections with an  Unidentified Initial Site of Infection | 16(5.25) | 6(10.91) | 0.191 |
| Laboratory indices |  |  |  |
| MPV, fL, median [IQR] | 10.000(9.3,11.0) | 10.300(9.4,11.3) | 0.337 |
| NEU, ×10^9^/L, median [IQR] | 9.240(6.4,13.4) | 9.000(5.2,12.0) | 0.295 |
| GGT, IU/L, median [IQR] | 42.000(20.1,88.7) | 39.000(25.0,71.9) | 0.827 |
| ALP, IU/L, median [IQR] | 67.000(51.5,96.0) | 70.000(50.0,97.0) | 0.777 |
| GLB, g/L, median [IQR] | 24.700(20.8,28.1) | 25.200(21.0,31.4) | 0.14 |
| TC, mmol/L, median [IQR] | 3.240(2.4,4.1) | 3.135(2.1,3.8) | 0.185 |
| TG, mmol/L, median [IQR] | 1.245(0.9,1.9) | 1.280(0.8,1.8) | 0.908 |
| GLU, mmol/L, median [IQR] | 7.300(5.4,9.6) | 7.790(5.4,8.9) | 0.835 |
| UA, umol/L, median [IQR] | 281.000(186.5,409.0) | 303.000(185.0,479.0) | 0.34 |
| BUN, mmol/L, median [IQR] | 8.070(5.7,12.3) | 10.840(6.2,18.9) | 0.072 |
| K, mmol/L, mean ±SD | 3.800(3.4,4.3) | 3.900(3.3,4.3) | 0.945 |
| * *p*<0.05 ** *p*<0.01  Data are number (%), mean ± standard deviation or median [interquartile range].  Abbreviations: MPV: mean platelet volume; NEU: neutrophil count; GGT: γ-glutamyl transferase; ALP: alkaline phosphatase; TC: total cholesterol; TG: triglycerides; GLU: glucose; UA: uric acid; BUN: blood urea nitrogen; K^+^: potassium. | | | |
